# C-Reactive Protein Is Not the Driver Factor in Ulcerative Colitis

**DOI:** 10.1155/2024/1386147

**Published:** 2024-10-01

**Authors:** Zhong-Bo Ge, Xin-Yun Zhang, Chun-Miao Zhang, Tao-Tao Xu, Si-Yi Li, Meng-Xiao Wei, Xin-Yuan Ding, Cai-Juan Bai, Han Wang, Hai-Hong Zhou, Ming-Yu Wang

**Affiliations:** ^1^ MOE Key Laboratory of Cell Activities and Stress Adaptations School of Life Sciences Lanzhou University, Lanzhou, Gansu 730000, China; ^2^ NHC Key Laboratory of Diagnosis and Therapy of Gastrointestinal Tumour The Institute of Clinical Research and Translational Medicine Gansu Provincial Hospital, Lanzhou, China; ^3^ Department of Blood Transfusion The First Hospital of Lanzhou University, Lanzhou, Gansu 730000, China; ^4^ Translational Medicine Research Centre Gansu Provincial Cancer Hospital, Lanzhou 730050, China

**Keywords:** C-reactive protein, CRP-deficient mice, ulcerative colitis

## Abstract

**Purpose**: C-reactive protein (CRP) functions as a nonspecific marker in various inflammatory disorders, particularly in evaluating the efficacy of pharmacological treatments in patients with ulcerative colitis. The existing body of evidence does not offer adequate support for the direct implication of CRP in modulating the advancement of ulcerative colitis.

**Methods**: Our study employed a rigorous mouse model. An ulcerative colitis mouse model was established by subjecting CRP-deficient mice to dextran sulfate sodium (DSS) treatment. The phenotype of the mice, which encompassed parameters such as body weight, colon length, and spleen weight, was meticulously evaluated. Additionally, various physiological and biochemical indicators were assessed, including colon histopathology, expression levels of inflammatory factors, and staining of the intestinal mucus layer.

**Results:** The absence of CRP did not significantly affect the phenotype, physiological characteristics, and biochemical indices in a mouse model of ulcerative colitis compared to mice with wild-type CRP. Additionally, eliminating intestinal bacteria flora interference through antibiotic treatment revealed that mice lacking CRP did not demonstrate any notable variations in the ulcerative colitis model. Meanwhile, the survival rate of mice lacking CRP did not exhibit a statistically significant difference compared to wild-type mice.

**Conclusion:** The results of our study suggest that CRP may not directly mediate ulcerative colitis. Instead, it is more likely to be a bystander that is present alongside with elevated inflammatory factors. Further investigation is warranted to determine the precise role of CRP in humans, given the significant limitations associated with the use of mouse models.

## 1. Introduction

C-reactive protein (CRP) is a well-known acute-phase protein, and its plasma levels rapidly increase in response to tissue injury or infection. As a result, it is widely recognized as a nonspecific indicator of inflammatory response [1–3]. CRP also assumes a crucial regulatory function in innate and acquired immunity, indicating its direct involvement in numerous chronic and acute inflammation-related disorders [4–6]. Ulcerative colitis (UC) is an inflammatory bowel disease that exhibits a rising global incidence with each passing year. It has been reported that there is a significant correlation between plasma CRP levels and the prognosis of various drugs used to treat UC [7–15]. Nevertheless, the direct involvement of CRP in regulating the progression of UC remains elusive. This study was aimed at elucidating the causal relationship between CRP and UC in a mouse model, intending to offer theoretical guidance for future research and treatment strategies for UC.

The association between plasma CRP levels and the occurrence and prognosis of UC merely establishes a correlation between the variables, making it challenging to discern a causal relationship. Although there are some differences in the acute response of CRP between humans and mice, the final functional roles of CRP from both species are primarily similar [16]. Hence, in the present study, we utilized CRP-deficient mice to establish the UC model. By assessing the phenotype and various physiological and biochemical indicators, CRP-deficient mice exposed to UC did not demonstrate any notable changes.

## 2. Material and Methods

### 2.1. Management of Animal Study

Wild-type C57BL/6J were obtained from the animal center of Lanzhou University, while CRP-deficient mice with C57BL/6J backgrounds were generously provided by Prof. Yi Wu [17]. The experimental mouse usage protocol had been approved by the Institution of Animal Care and Use Committee (approval: EAF2021009, Lanzhou University, China). In brief, the mice were housed with a 12-h light/dark cycle (light: 7:00–19:00), with 40%–50% relative humidity at 21°C–22°C, and with free access to food and water in an SPF-grade animal center from School of Life Sciences at Lanzhou University.

### 2.2. Mouse Model

For the UC mouse model, wild-type C57BL/6J or CRP-deficient male mice aged 8–10 weeks were randomly grouped and subjected to the induction of UC using a 2% dextran sulfate sodium (DSS) solution in their drinking water every 2 days for 7–10 days. The mice's weight was measured during the experiments. At the endpoint of the experiments, the mice were euthanized using carbon dioxide. Then, spleen weight and colon length were measured. Colon samples were resected for histological assay and Western blot. Serum was collected from the orbital sinus for enzyme-linked immunosorbent assay (ELISA). Among these experiments, the causal roles of CRP were assayed in CRP-deficient mice and wild-type mice as controls. The number of mice for each experiment is variable according to the supply capacity of mice, with at least five mice for each group to reach adequate statistical power.

According to the reported method, a 5% DSS solution was used for survival analysis [18]. The experiment was terminated until the total number of mice killed reached 90%, and the remaining mice were euthanized using carbon dioxide. The survival function was estimated using the Kaplan–Meier method.

### 2.3. Antibiotic Experiments

For the antibiotic experiments, a concentration of 1 g/L ampicillin was added to the drinking water [19] 2 weeks prior to the commencement of the study. The effect of intestinal flora depletion was assayed through fecal microbiota culture, according to a previous report [20]. In brief, the collected fecal samples from the mice were suspended and diluted 2 × 10^5^-fold with sterile water. The diluent was inoculated, spread on a nutrient agar plate (0.5% peptone, 0.3% beef extract/yeast extract, 1.5% agar, and 0.5% NaCl), and aerobically incubated at 30°C for 24 h. The bacteria colonies in the nutrient agar plate were counted. The intestinal flora–depleted mice of wild-type or CRP-deficient C57BL/6J male mice were subjected to the induction of UC. The body weight, colon length, and spleen index were recorded during the experiment.

### 2.4. Hematoxylin and Eosin (HE) and PAS Staining

For the HE staining procedure, colon samples from mice were resected and subsequently fixed in a 4% paraformaldehyde solution overnight at 4°C. Dehydration and paraffin embedding procedures were carried out following established protocols. The paraffin-embedded tissues were rehydrated sequentially using xylene and ethanol, followed by staining with HE. For the PAS staining procedure, the tissues were fixed using a Carnoy fixative as an alternative to paraformaldehyde. The remaining steps were analogous to the HE staining procedure, including an additional step for PAS staining.

### 2.5. Gene Expression Analysis (Western Blot and ELISA)

The UC colon samples and serum were derived from mouse models with wild-type mice as controls. The macrodissected colon samples and serum (derived from the orbital sinus) were frozen in liquid nitrogen and stored in a refrigerator at −80° until detection. Total protein was extracted utilizing lysis buffer (50 mM Tris-HCl, 150 mM NaCl, 1 mM EDTA, and 1% SDS). Thirty micrograms of total protein quantified through the BCA method was loaded, which was performed according to the standard Western blot protocol. The antibodies against mouse Muc2 or Tff3 were purchased from ABclonal Technology (catalog: A4767 for Muc2) and Abcam (catalog: ab30042 for Tff3), respectively. Tubulin was used as the control (ABclonal Technology, catalog: WX06622).

The concentration of IL-1*β*, IL-6, and IL-17 in serum was evaluated through ELISA according to the instructions of the ELISA kit (Shanghai Kexing Trading, Co., Ltd.; catalog: F2040-A for IL-1*β*, F2163-A for IL-6, and F2170-A for IL-17).

### 2.6. Statistical Analysis

Data were presented as *means* ± *standard* *deviation* (SD). All experimental procedures were replicated a minimum of three times. The statistical analysis used the Student *t*-test in OriginPro software (OriginLab Corporation).

## 3. Results

### 3.1. CRP Deficiency Elicits a Comparable Response to That Observed in Wild-Type Mice With UC

**Figure 1:**
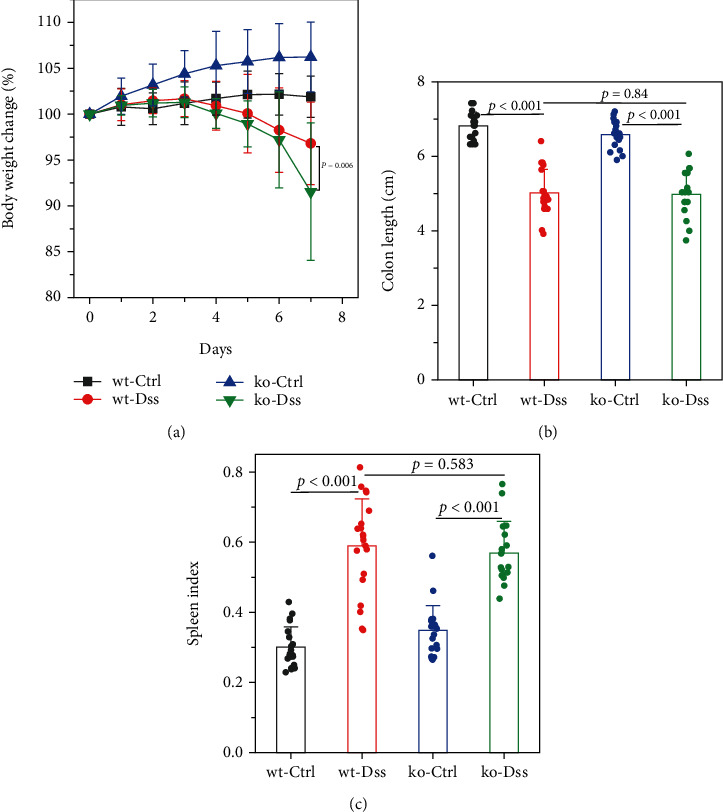
Mouse model had ulcerative colitis in wild-type and CRP-deficient mice. Ulcerative colitis was induced using 2% DSS (*n* = 20 for each group). (a) Body weight, (b) colon length, and (c) splenic index were measured. The splenic index indicates *splenic* *weight*∗1000/*mouse* *body* *weight*. The wt indicates the wild-type mice, and ko indicates the CRP-deficient mice.

Our study in a mouse model of UC induced with DSS revealed significant weight loss in both CRP-deficient and wild-type mice (Figure 1). Despite more pronounced weight loss in CRP-deficient mice (Figure 1(a)), more precise measures of colitis severity, like colon length (Figure 1(b)) and spleen index (Figure 1(c)), showed comparable results. These results suggested that the expression of CRP in mice did not significantly impact the progression of UC.

**Figure 2:**
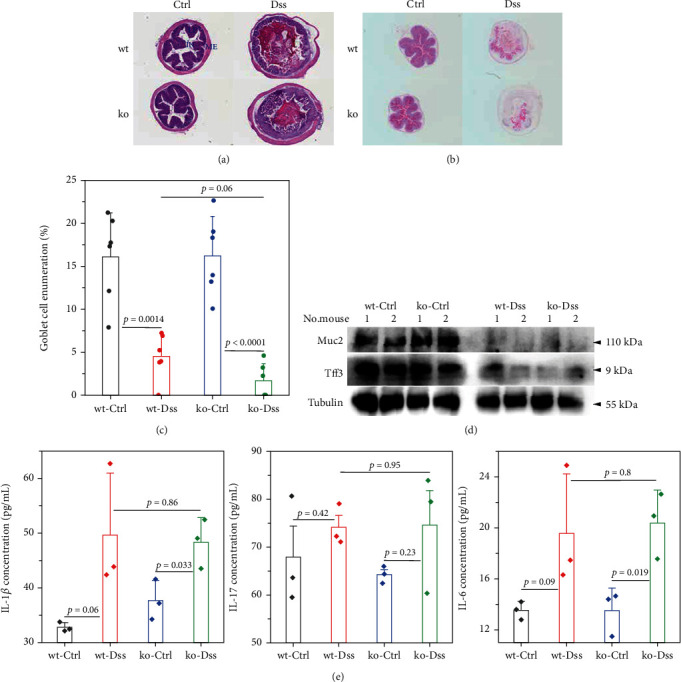
CRP deficiency did not affect the inflammatory indicators in ulcerative colitis. The colon samples derived from mouse models (Figure 1) were subjected to (a) HE and (b) PAS staining (LP: lamina propria; ME: muscular; IN: inclusion). The number of goblet cells was quantified using the ratio of goblet cells to intestinal villous epithelium from PAS staining (c, *n* = 6 mice for each group). The relative expressions of goblet cells' associated factors Muc2 and Tff3 were assayed using Western blot (d, *n* = 2 mice for each group). Inflammatory cytokines IL-6, IL-1*β*, and IL-17 were assayed using serum through ELISA (e, *n* = 3 mice for each group).

To thoroughly investigate the potential impact of CRP on UC, we conducted a series of meticulous histochemical staining experiments. Our findings indicated that the administration of DSS exacerbated the pathology associated with UC. However, we observed no significant variation in this pathology between subjects with or without CRP (Figure 2(a)). Meanwhile, applying PAS staining to detect goblet cells suggested that CRP might not be associated with the pathological alterations observed in UC (Figures 2(b) and 2(c)). In addition, the expression levels of mucosal markers muc2 and Tff3 were comparable between the CRP-deficient and control groups (Figure 2(d)). The etiology of UC has been extensively studied and widely acknowledged as a predominant influence of immune system dysregulation. The results demonstrated no significant difference in the circulating protein expression of major inflammatory factors (IL-1*β*, IL-6, IL-17) between CRP-deficient mice and wild-type mice (Figure 2(e)).

### 3.2. The Lack of Efficacy of CRP in UC Is Not Influenced by the Composition of the Intestinal Flora

**Figure 3:**
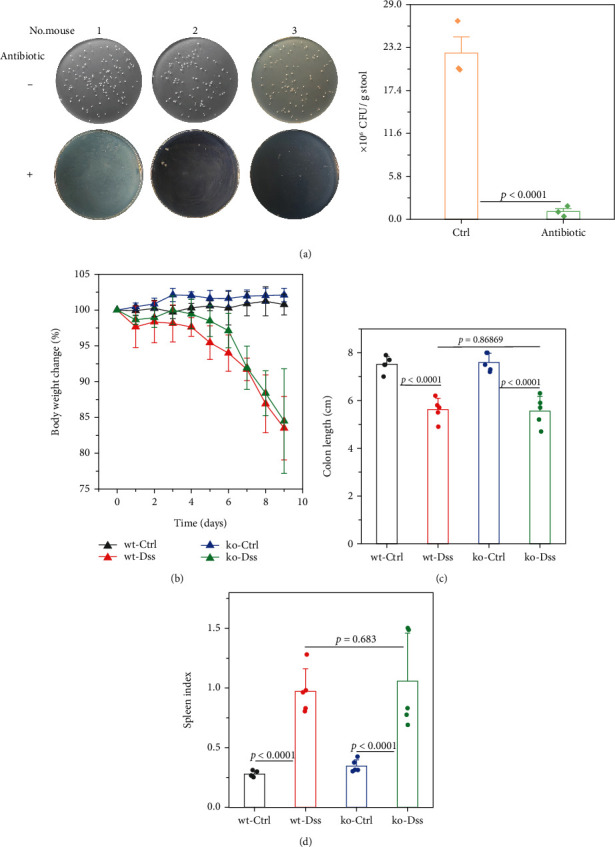
CRP presented as useless in ulcerative colitis regardless of the intestinal flora. Fecal microbiota cultures from wild-type mice (treated with or without antibiotics) were used to evaluate the effect of intestinal flora depletion (a, *n* = 3 mice for each group). The wild-type and CRP-deficient mice (with or without DSS treatment, *n* = 5 for each group) were all subjected to antibiotic treatment. The (b) weight changes, (c) colon length, and (d) spleen index were measured.

**Figure 4:**
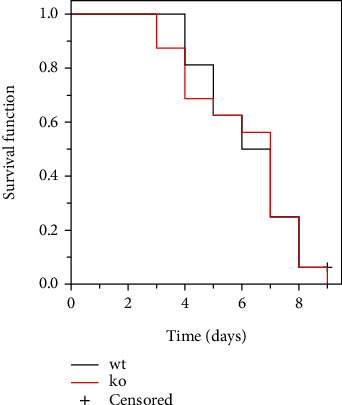
Survival analysis of CRP-deficient mice in ulcerative colitis. The survival function of wild-type and CRP-deficient mice (*n* = 16 for each group) induced by 5% DSS was estimated using the Kaplan–Meier method.

Inflammatory bowel disease typically results in the disturbance of the intestinal mucosal barrier system [21–26]. The intestinal microbiota play a significant role in the development and progression of inflammatory bowel disease through the disordered intestinal barrier system [27]. Additionally, reports indicate that CRP can bind to bacteria, serving as a significant opsonizing agent [28, 29]. In our comprehensive study, we provided additional clarification on the potential functional contributions of CRP by conducting a depletion of the intestinal flora using antibiotics (the depletion effect up to 95%, as shown in Figure 3(a)). The findings indicated that the mice, with or without CRP, did not yield a significant difference in UC (Figures 3(b), 3(c), and 3(d)). In a model of UC induced by high concentrations of DSS to assess the survival function, no significant differences were observed between CRP-deficient and wild-type mice (Figure 4). Altogether, our study suggests that CRP is more likely to be a bystander in UC.

## 4. Discussion

CRP is frequently employed as a diagnostic indicator for numerous diseases in clinics. Numerous recent reports suggest that CRP functions as a marker and plays a direct role in disease progression, making it a potential therapeutic target for specific diseases [2]. Clinical evidence indicates a significant correlation between CRP and the development and prognosis of UC [7–15]. However, it remains challenging to ascertain whether CRP directly contributes to the progression of colitis or solely serves as a molecular marker. This difficulty arises from the need for an appropriate model for investigation. Researchers have utilized single-nucleotide polymorphisms (SNPs) that impact CRP levels to establish the causal association between CRP and the development of cancer [30–34] and cardiovascular diseases [35–37]. SNPs exhibit a restricted impact on plasma CRP levels, particularly during the acute phase. UC is characterized by an acute inflammatory response, indicating that the precise causal relationship between CRP and UC may not be reliably determined solely by identifying SNPs.

Researchers have raised valid concerns about using mouse models to study the role of CRP, primarily due to observed differences in mouse CRP levels compared to humans during acute-phase responses [38]. Recent studies have suggested that the underestimation of mouse CRP expression levels may be due to inappropriate detection antibodies [39]. However, a fundamental structural and functional similarity exists between mouse and human CRP, indicating that both function similarly in the mouse model [16]. Hence, we used mouse models as a tool to investigate the functionality of CRP. Our study, which utilizes a mouse strain with a complete knockout of CRP, is aimed at investigating the causal role of CRP in UC, thereby enhancing the reliability of the results. The findings of this study suggest that CRP is more likely to be a bystander in the development of UC rather than a causative factor or mediator of the disease.

CRP, the primary acute-phase protein, is predominantly expressed by the liver and secreted into the circulatory system [40]. In UC, localized colitis triggers the production of inflammatory factors such as IL-6 and IL-1*β*, which significantly stimulate the liver to produce CRP, thereby elevating CRP levels in UC patients. This established association between CRP and UC allows CRP to predict the pathogenesis and prognosis of the disease. It guides medication decisions, making it a crucial diagnostic tool in the clinical setting. Despite its limitations, the role of CRP extends beyond being a mere marker. Its functions and implications are still largely unknown and require further investigation to be fully understood.

## Data Availability

The data supporting this study's findings are available in this paper or on request from the corresponding authors.
